# Supplementary material for: ERAD deficiency promotes mitochondrial dysfunction and transcriptional rewiring in human hepatic cells
Source: J Biol Chem. 2021 Jan 13;295(49):16743–53. doi: 10.1074/jbc.RA120.013987 (PMC7864069; doi:10.1074/jbc.RA120.013987)
Supplement: Supplementary file 1 [file mmc1.pdf]

SUPPORTING DATA

ERAD Deficiency Promotes Mitochondrial Dysfunction and Transcriptional Rewiring in Human Hepatic Cells

Cell culture, morphological and functional analysis of mitochondria

Wild type and Sel1L-deficient HepG2 cells were cultured in DMEM medium supplemented with 15% FBS, 100 U/ml penicillin and 100 μg/ml streptomycin at 37℃ in an humidified CO_2 incubator. For visualization of mitochondria, a plasmid expressing mitochondria-targeted green fluorescent protein (GFP), pmtGFP, was transfected into HepG2 cells using Lipofectamine 3000. GFP images were acquired with a FluoviewFV1000 Olympus confocal microscope. For determination of mitochondrial Transmembrane Potential (MTP), HepG2 cells were stained with JC-1 dye (Abcam) and analyzed by fluorescence activated cell sorting (FACS). For assessment of mitochondrial function, the oxygen consumption rate (OCR) and extracellular acidification rate (ECAR) of HepG2 cells were assayed using the XF Cell Mito Stress Test Kit and XF Glycolysis Stress Test Kit, respectively, coupled with the XF24-3 Extracellular Flux Assay Kit. All OCR and ECAR measurements were normalized to protein content.

Fig S1

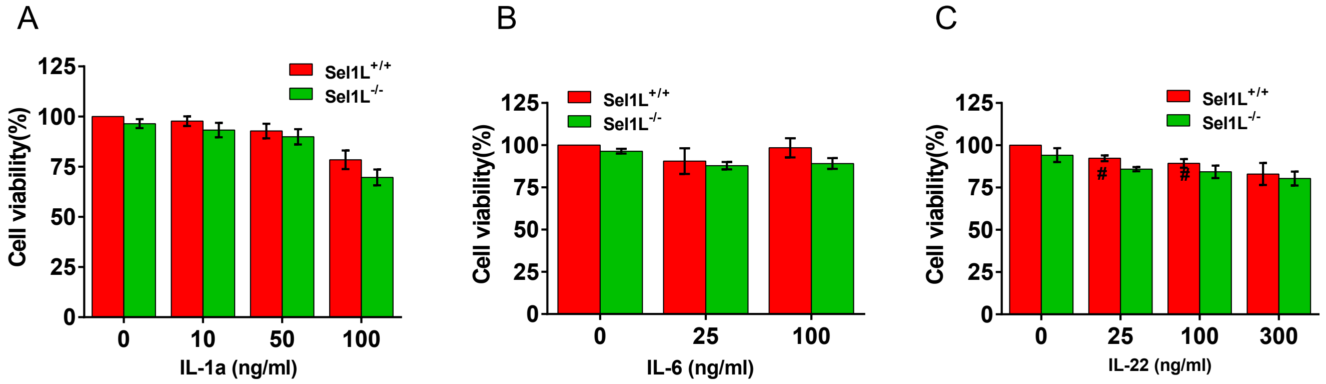
A
B
C
Cell viability(%)
Sel1L+/+
Sel1L-/-
IL-1a (ng/ml)
IL-6 (ng/ml)
IL-22 (ng/ml)
0
10
50
100
25
300
125
75
#

Fig S2

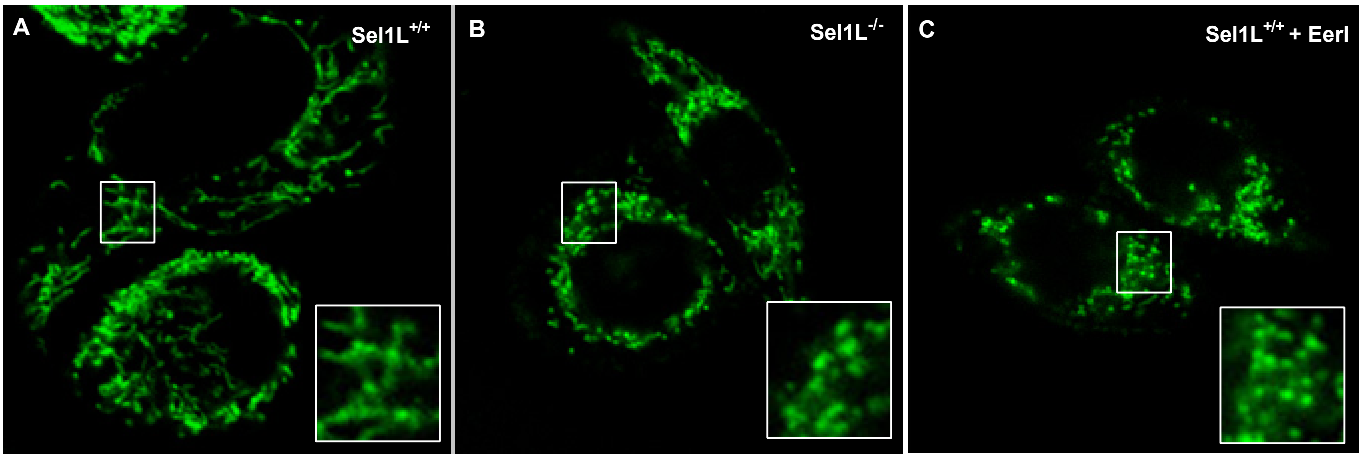

Fig S3

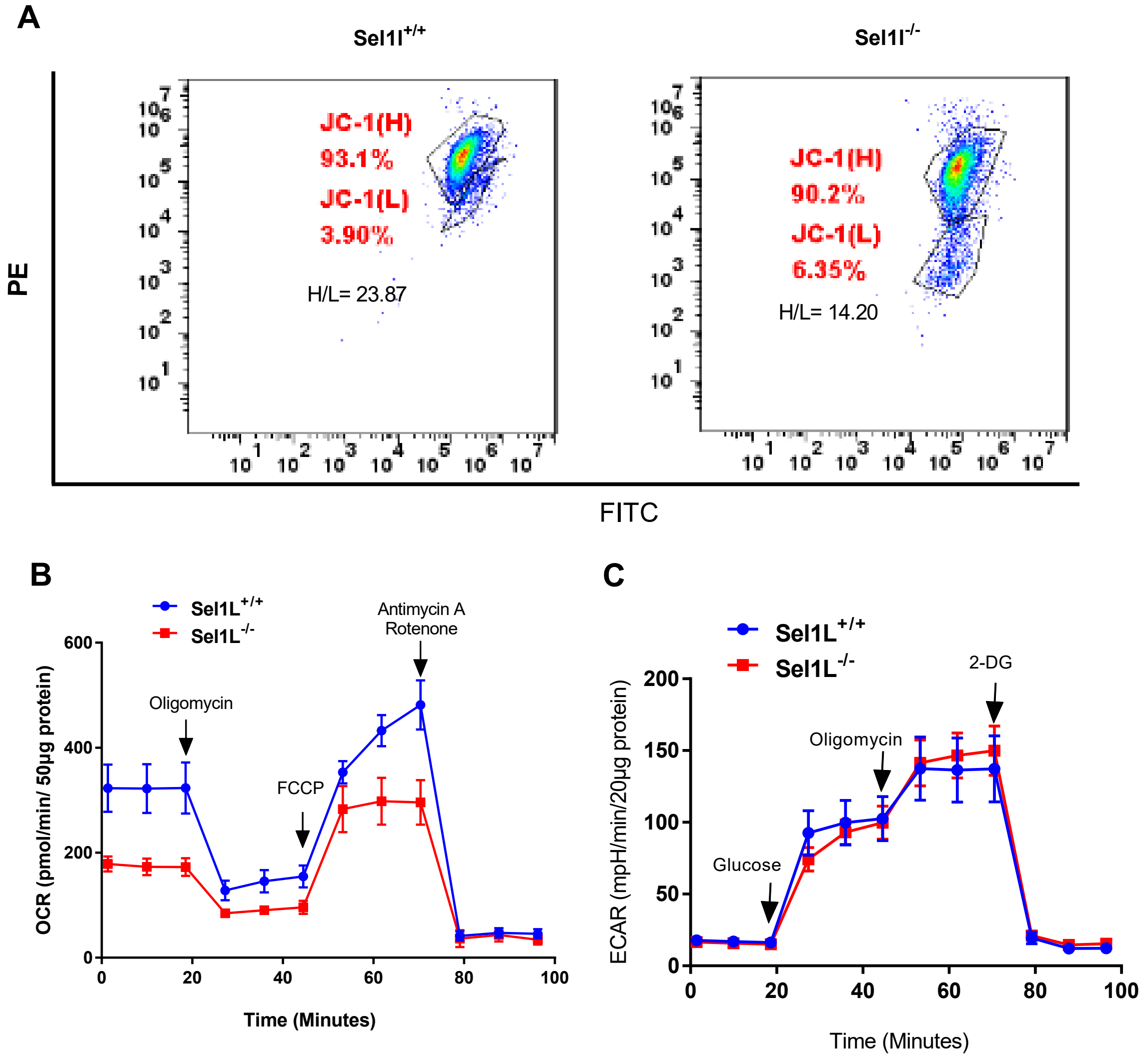
A
Sel1l+/+
Sel1l-/-
JC-1(H)
93.1%
JC-1(L)
3.90%
H/L= 23.87
JC-1(H)
90.2%
JC-1(L)
6.35%
H/L= 14.20
PE
FITC
B
Sel1L+/+
Sel1L-/-
Oligomycin
FCCP
Antimycin A
Rotenone
OCR (pmol/min/ 50µg protein)
Time (Minutes)
C
Sel1L+/+
Sel1L-/-
Glucose
Oligomycin
2-DG
ECAR (mpH/min/20µg protein)
Time (Minutes)

Figure S1 Effects of IL-1α, IL-6 and IL-22 on the viability of Sel1L$^{+/+}$ and Sel1L$^{-/-}$ cells. Sel1L$^{+/+}$ and Sel1L$^{-/-}$ cells were treated for 24 hours with the indicated concentrations of IL-1α, IL-6 and IL-22, and the treated cells were analyzed by MTT assay.

Figure S2 Fluorescence**Mitochondrial morphology and glycolysis stress test profiles of Sel1L$^{+/+}$ and Sel1L$^{-/-}$ cells**. (A-C) Fluorescent images of Sel1L$^{+/+}$, Sel1L$^{-/-}$, and EerI-treated Sel1L$^{+/+}$ cells after transfection with a plasmid expressing mitochondria-targeted green fluorescent protein (GFP) reporter. Inset at the bottom right of each panel represents a magnified view of the dash line marked area.

Figure S3 Mitochondrial membrane potential and bioenergetic profiles of Sel1L$^{+/+}$ and Sel1L$^{-/-}$ cells. (A) FITC and PE dual channel FACS analysis of Sel1L$^{+/+}$ and Sel1L$^{-/-}$ cells stained with JC-1. H/L ratio represents mitochondrial membrane potential. (B) Oxygen consumption rate (OCR) (basal or in the presence of oligomycin, FCCP, Antimycin A and Rotenone). (C) Extracellular acidification (ECAR) rate. Compounds sequentially injected during the stress test include: glucose, oligomycin and 2-deoxy-glucose (2-DG), which measures glycolysis, glycolytic capacity and allows calculation of glycolytic reserve and nonglycolytic acidification.
